# Establishment of a novel ferroptosis-related lncRNA pair prognostic model in colon adenocarcinoma

**DOI:** 10.18632/aging.203599

**Published:** 2021-10-05

**Authors:** Hong Li, Lili Liu, Tianyi Huang, Ming Jin, Zhen Zheng, Hui Zhang, Meng Ye, Kaitai Liu

**Affiliations:** 1 Department of General Surgery, The Affiliated Lihuili Hospital, Ningbo University, Ningbo, China; 2 Department of Medical Oncology, The Second Affiliated Hospital of Dalian Medical University, Dalian, China; 3 Department of Radiation Oncology, The Affiliated Lihuili Hospital, Ningbo University, Ningbo, China; 4 Department of Oncology and Hematology, The Affiliated Hospital of Medical School of Ningbo University, Ningbo, China

**Keywords:** colon adenocarcinoma, ferroptosis, lncRNA pairs, The Cancer Genome Atlas, prognostic model

## Abstract

Long non-coding RNAs (lncRNAs) have been reported to be prognostic factors for cancer. Ferroptosis is an iron-dependent process of programmed cell death. Here, we established a ferroptosis-related lncRNA (frlncRNA) pair signature and revealed its prognostic value in colon adenocarcinoma (COAD) by analyzing the data from The Cancer Genome Atlas (TCGA). FrlncRNAs were identified based on co-expression analysis using the Pearson correlation. Differentially expressed frlncRNAs (DEfrlncRNAs) were recognized and paired, followed by prognostic assessment using univariate Cox regression analysis. The least absolute shrinkage and selection operator (LASSO) penalized Cox analysis was used to determine and construct a risk score prognostic model, by which the receiver operating characteristic (ROC) curves for predicting the overall survival (OS) were conducted. Following the evaluation of whether it was an independent prognostic factor, correlations between the risk score model and clinicopathological characteristics, hypoxia- and immune-related factors, and somatic variants were investigated. In total, 148 DEfrlncRNA pairs were identified, 25 of which were involved in a risk score prognostic signature. The area under ROC curves (AUCs) representing the predictive effect for 1-, 3-, and 5-year survival rates were 0.860, 0.885, and 0.934, respectively. The risk score model was confirmed as an independent prognostic factor and was significantly superior to the clinicopathological characteristics. Correlation analyses showed disparities in clinicopathological characteristics, hypoxia- and immune-related factors, and somatic variants, as well as specific signaling pathways between high- and low-risk groups. The novel risk score prognostic model constructed by pairing DEfrlncRNAs showed promising clinical prediction value in COAD.

## INTRODUCTION

Colon cancer (CC) is one of the most frequently diagnosed malignant tumors worldwide and is a leading cause of cancer-related deaths in China [1–2]. Approximately 80%–90% of CC cases are colon adenocarcinoma (COAD) based on the pathologic classification [3]. In general, 20%–25% of CC patients are diagnosed with unresectable metastatic disease and have a poor prognosis [4]. In recent years, the 5-year over survival (OS) rate of CC patients with local stage disease was 90.3%, while that of patients with distant metastases was 12.5% [5]. Thus, developing promising prognostic-related risk assessment signatures is of great clinical significance in the management of colon cancer.

Long non-coding RNAs (lncRNAs), which are a subset of RNAs more than 200 nucleotides in length, account for approximately 80% of the human transcriptome [6]. LncRNAs do not code proteins but exert biological functions by regulating gene expression [7]. Studies have reported that lncRNAs are involved in the malignant phenotypes of various cancers, including proliferation, invasion, and treatment resistance [8]. Evidence has shown abnormal lncRNA expression in tumor samples compared to the corresponding adjacent normal tissues [9, 10]. In addition, studies have demonstrated that lncRNAs contribute to tumorigenesis not only through physiological and biochemical cellular processes, such as ferroptosis, antioxidant capacity, apoptosis, and autophagy, but also by altering the immune microenvironment in cancers [11–13]. Ferroptosis is an iron-dependent cell death first proposed by Dixon in 2012 and is characterized by an intracellular iron-dependent accumulation of reactive oxygen species (ROS) and lipid peroxidation [14]. It is a new form of cell death that differs from apoptosis and autophagy [15, 16]. Recent studies have revealed that ferroptosis is a critical factor in metabolism, redox biology, and cell death and has been gradually confirmed as a novel feature for cancer therapy, particularly for those resistant to traditional therapies [17, 18].

Previous studies have reported lncRNA prognostic signatures that have promising predictive and prognostic values in cancer. Tang et al. developed a signature that included 25 differentially expressed ferroptosis-related lncRNAs to predict the prognosis of head and neck squamous cell carcinoma (HNSCC) [19]. The area under the receiver operator characteristic (ROC) curve (AUC) of the lncRNA signature was 0.782, showing a promising prediction value for HNSCC. Moreover, Shen et al. identified and validated an immune-related lncRNA prognostic signature for breast cancer. In this prior study, the prognostic signature comprised 11 lncRNAs and was associated with the infiltration of immune cell subtypes [20]. Furthermore, Soudeh Ghafouri-Fard et al. reviewed the lncRNA signature in gastric cancer based on the available literature and concluded that these transcripts deserve further evaluation as therapeutic targets in gastric cancer [21]. In addition, Wei et al. constructed an autophagy-related lncRNA signature comprising eight lncRNAs and predicted unfavorable prognosis in colorectal cancer. The AUC of this signature was 0.689, indicating that the risk model was effective [22]. Zeng et al. established a differentially expressed lncRNA signature to evaluate the outcome of patients with colorectal cancer. They found that 20 lncRNAs closely related to OS in patients with COAD, four of which were involved in a prognostic model, could serve as an independent factor for survival in COAD [23]. Although the lncRNA signature showed promising prediction performance in cancer prognosis, it was not perfect. The signature required specific expression levels of selected lncRNAs, which should be normalized to reduce the batch effects between different testing platforms before clinical application. Additionally, Hong et al. utilized a novel modeling algorithm, pairing, and iteration to construct an immune-related lncRNA pair prognosis signature in human hepatocellular carcinoma [24]. The AUCs of 1-, 3-, and 5-year survival rates were 0.865, 0.851, and 0.904, respectively. However, there are few reports on lncRNA pair models for cancer prognostic prediction.

In the present study, we developed a novel ferroptosis-related lncRNA pair (frlncRNA pair) prognostic model for COAD. Correlations between the model and clinicopathological characteristics, hypoxia-, immune-related factors, and somatic variants were investigated.

## RESULTS

### Data characteristics

The expression data of 480 colon adenocarcinoma and 41 adjacent normal samples were included in the present study. The clinical information of patients (n=459) including age, gender, stage, T status, N status, and M status, are shown in Table 1. A total of 259 ferroptosis-related genes (frGenes) were downloaded from the FerrDb website (Supplementary Table 1). Overall, 896 lncRNAs were identified as ferroptosis-related lncRNAs (frlncRNAs) (Supplementary Table 2). Subsequently, 165 (including 158 upregulated and 7 downregulated) of these were identified as differentially expressed ferroptosis-related lncRNAs (DEfrlncRNAs) (Supplementary Table 3), which were visualized using a heatmap (Supplementary Figure 1) and a volcano plot (Supplementary Figure 2).

**Table 1:** The clinical characteristics of COAD patients in the TCGA dataset.

**Variables**	**Number of cases**
Total	459
Age (years)	
<60 / ≥60	126/333
Gender	
Male/Female	216/243
Stage	
I/II/III/IV/NA	76/177/129/65/11
T	
T0/T1/T2/T3/T4	1/11/78/313/56
N	
N0/N1/N2	270/106/83
M	
M0/M1/NA	337/65/57

### Establishment of a frlncRNA pair risk score prognostic model

**Figure 1:**
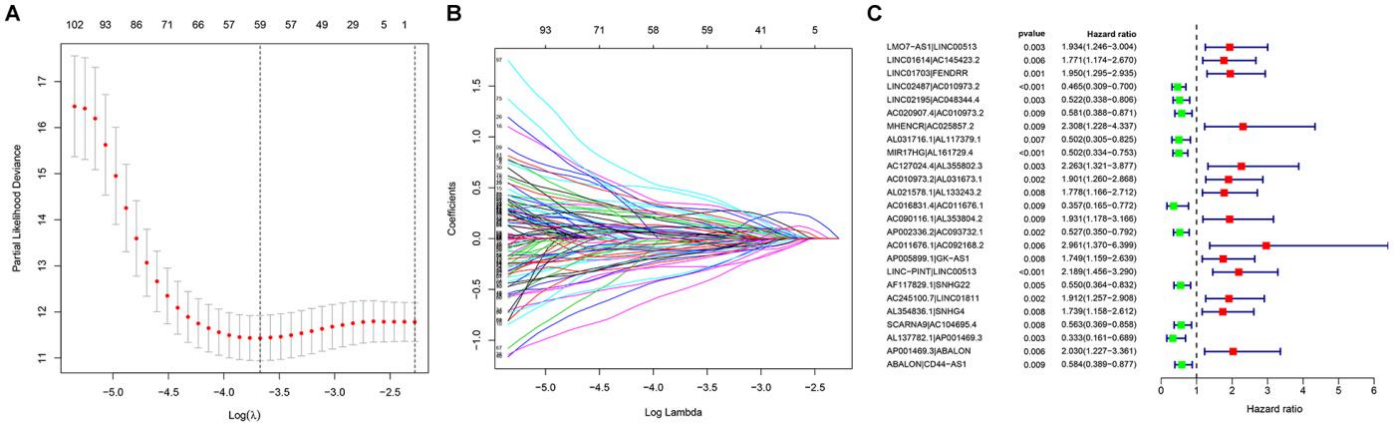
Establishment of a prognostic model based on (**A**, **B**) LASSO regression analysis; (**C**) Univariate Cox regression analysis.

**Figure 2:**
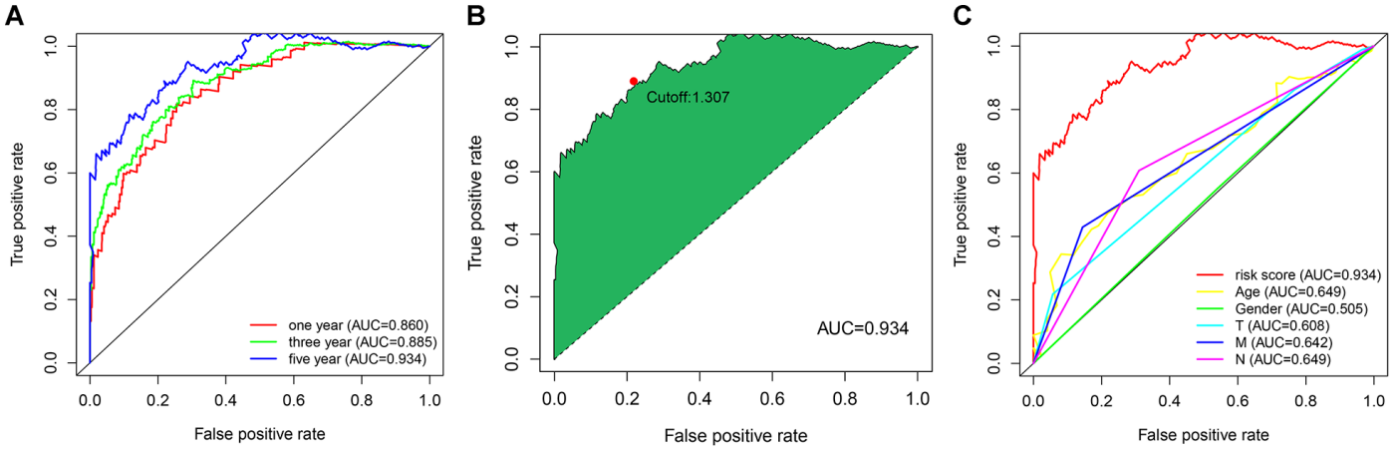
(**A**) The ROC curves for predicting the 1-, 3-, and 5-year OS; (**B**) identification of the maximum inflection point as the optimal cut-off value on the 5-year ROC curve; (**C**) comparison of the risk score model and clinicopathological characteristics in predicting the 5-year OS.

To explore a more objective prognostic evaluation model that did not require the specific expression values to be normalized, a 0-or-1–matrix of 7543 frlncRNA pairs was constructed (Supplementary Table 4). According to univariate Cox proportional hazards regression analyses, 148 frlncRNA pairs were considered to be prognostic-associated lncRNA pairs (Supplementary Table 5). In training cohort, after the least absolute shrinkage and selection operator (LASSO) regression analysis, a prognostic signature including 44 frlncRNA pairs was established (Supplementary Table 6). The AUC representing the predictive value of the risk score model for 1-year survival rate in training and validation cohort were 0.904 and 0.728, respectively (Supplementary Figure 3A, 3B). Survival analyses showed significant difference between the high- and low-risk groups both in training and validation cohort (Supplementary Figure 3C, 3D). Subsequently, we constructed a risk score model based on the data of entire cohort (Figure 1). The list of 25 frlncRNA pairs and their corresponding calculation coefficients are shown in Table 2. The AUCs for 1-, 3-, and 5-year survival rates were 0.860, 0.885, and 0.934, respectively (Figure 2A). Furthermore, we identified the maximum inflection point of 1.307 as the optimal cut-off point on the 5-year receiver operator characteristic (ROC) curve (Figure 2B). Moreover, our results showed that the risk score model was significantly superior to the common clinicopathological characteristics, including age, gender, T status, N status, and M status in predicting the OS of COAD patients (Figure 2C).

**Table 2:** The list of lncRNA pairs and corresponding calculation coefficients.

**LncRNA pair**	**Coefficient**
LMO7-AS1|LINC00513	0.493078
LINC01614|AC145423.2	0.746247
LINC01703|FENDRR	0.375638
LINC02487|AC010973.2	-0.47787
LINC02195|AC048344.4	-0.89094
AC020907.4|AC010973.2	-0.67771
MHENCR|AC025857.2	0.728441
AL031716.1|AL117379.1	-0.76209
MIR17HG|AL161729.4	-0.64218
AC127024.4|AL355802.3	0.774316
AC010973.2|AL031673.1	0.521472
AL021578.1|AL133243.2	0.426426
AC016831.4|AC011676.1	-0.94739
AC090116.1|AL353804.2	0.49057
AP002336.2|AC093732.1	-0.56376
AC011676.1|AC092168.2	0.835864
AP005899.1|GK-AS1	0.378791
LINC-PINT|LINC00513	0.605353
AF117829.1|SNHG22	-0.57948
AC245100.7|LINC01811	0.77997
AL354836.1|SNHG4	0.551547
SCARNA9|AC104695.4	-0.39001
AL137782.1|AP001469.3	-0.95082
AP001469.3|ABALON	0.598743
ABALON|CD44-AS1	-0.64299

### Predictive assessment and clinical correlation of the prognostic model

**Figure 3:**
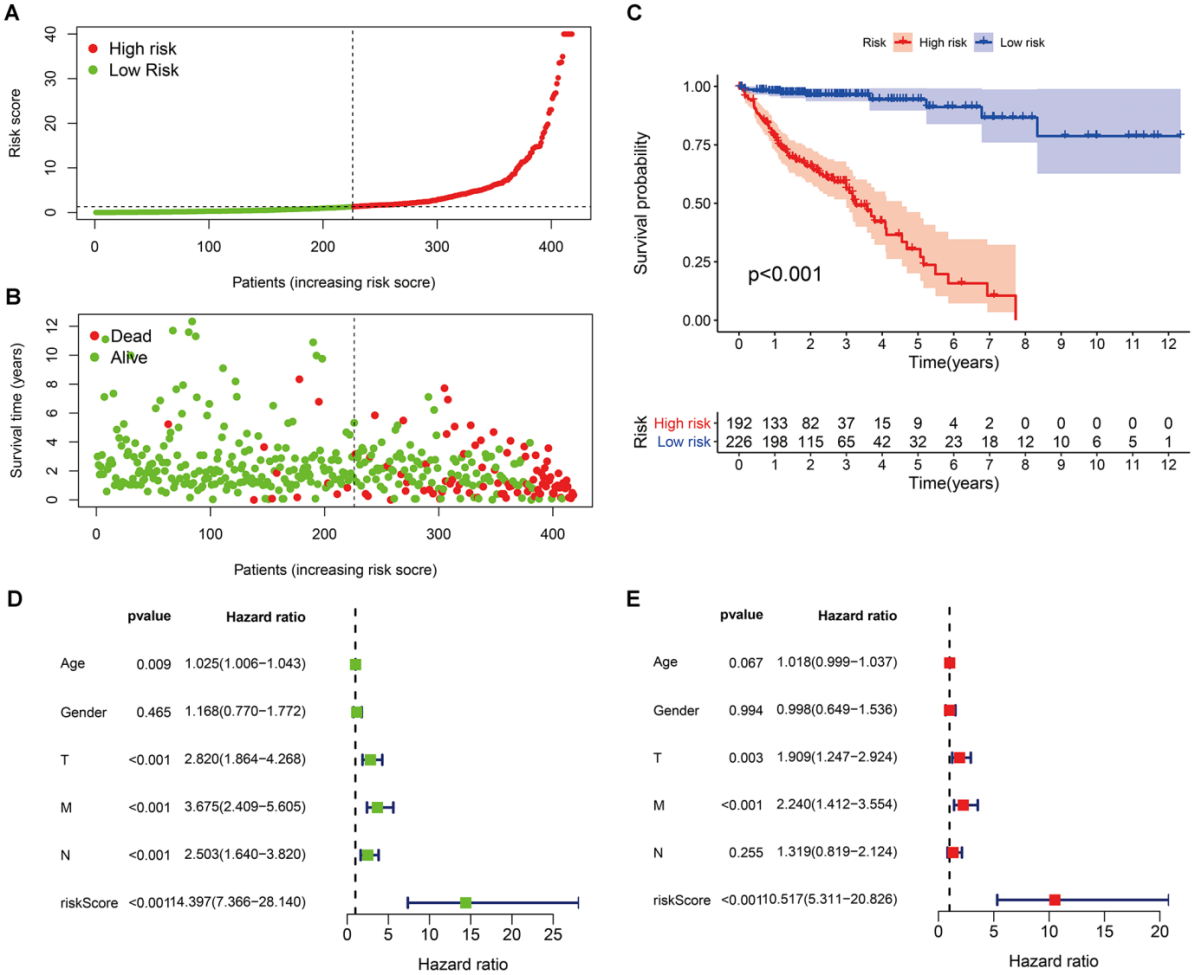
Risk scores (**A**) and survival outcomes (**B**) of each patient; (**C**) survival curves of high-risk group and low-risk group patients; (**D**) univariate and (**E**) multivariate Cox regression analyses of the risk score model and clinicopathological characteristics.

**Figure 4:**
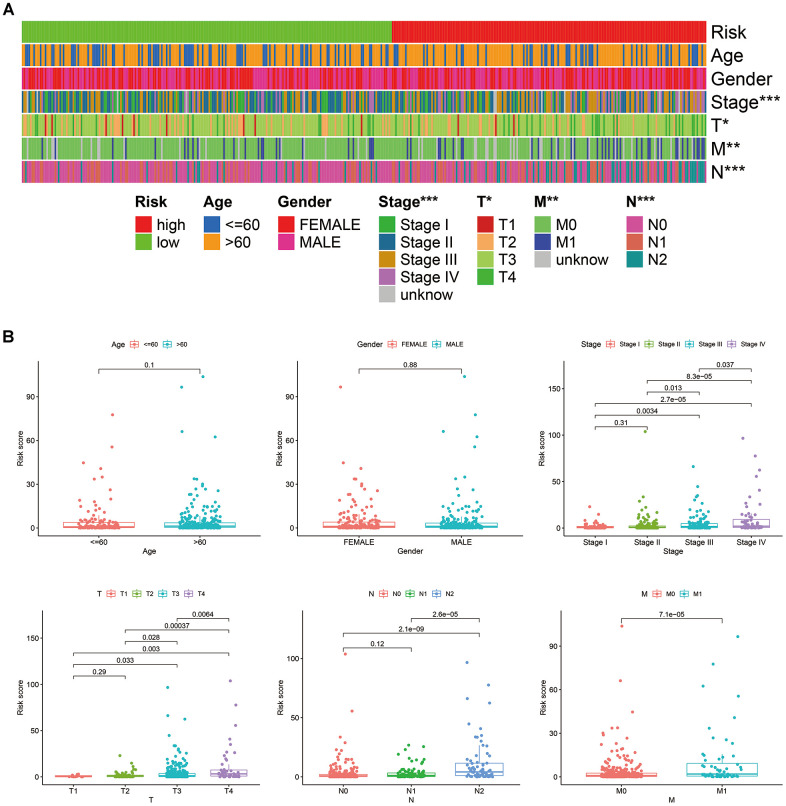
Correlations between the risk score model and clinicopathological characteristics, represented by a heatmap (**A**), and box diagrams (**B**).

**Figure 5:**
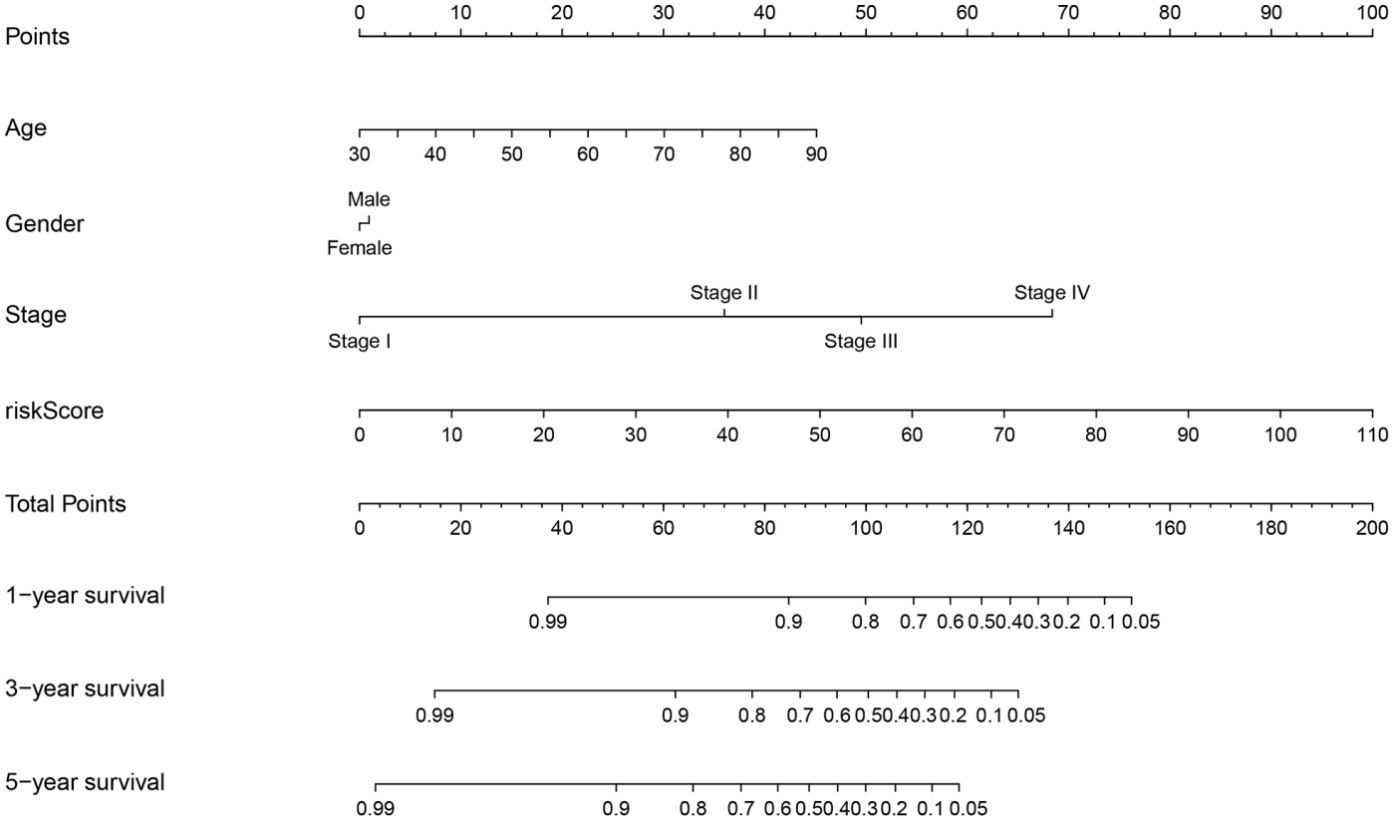
Prognostic nomogram incorporating the risk score model and clinicopathological characteristics.

According to the cut-off point recognized previously, 226 patients were classified into the low-risk group and 192 into the high-risk group (Supplementary Table 7). The risk assessment model for prognosis prediction demonstrated that the number of deaths increased with an increase in the risk score (Figure 3A, 3B). Survival analysis revealed that the high-risk group had significantly worse OS than low-risk group (Figure 3C). Age, T status, N status, M status, and risk score model were identified as significant risk factors in the univariate analysis (all *P*<0.01) (Figure 3D). The risk score model, T status and M status were confirmed as independent prognostic factors by multivariate analysis (all *P*<0.01) (Figure 3E). Furthermore, our results showed that the risk score model was significantly related to T status, N status, M status, and stage (Figure 4). Moreover, an accurate prognostic nomogram incorporating the risk score model and common clinicopathological characteristics was established for predicting 1-, 3-, and 5-year OS probability, which might be well applied in the clinical evaluation of COAD patients (Figure 5).

### Functional enrichment analyses of differently expressed frGenes (DEfrGenes)

**Figure 6:**
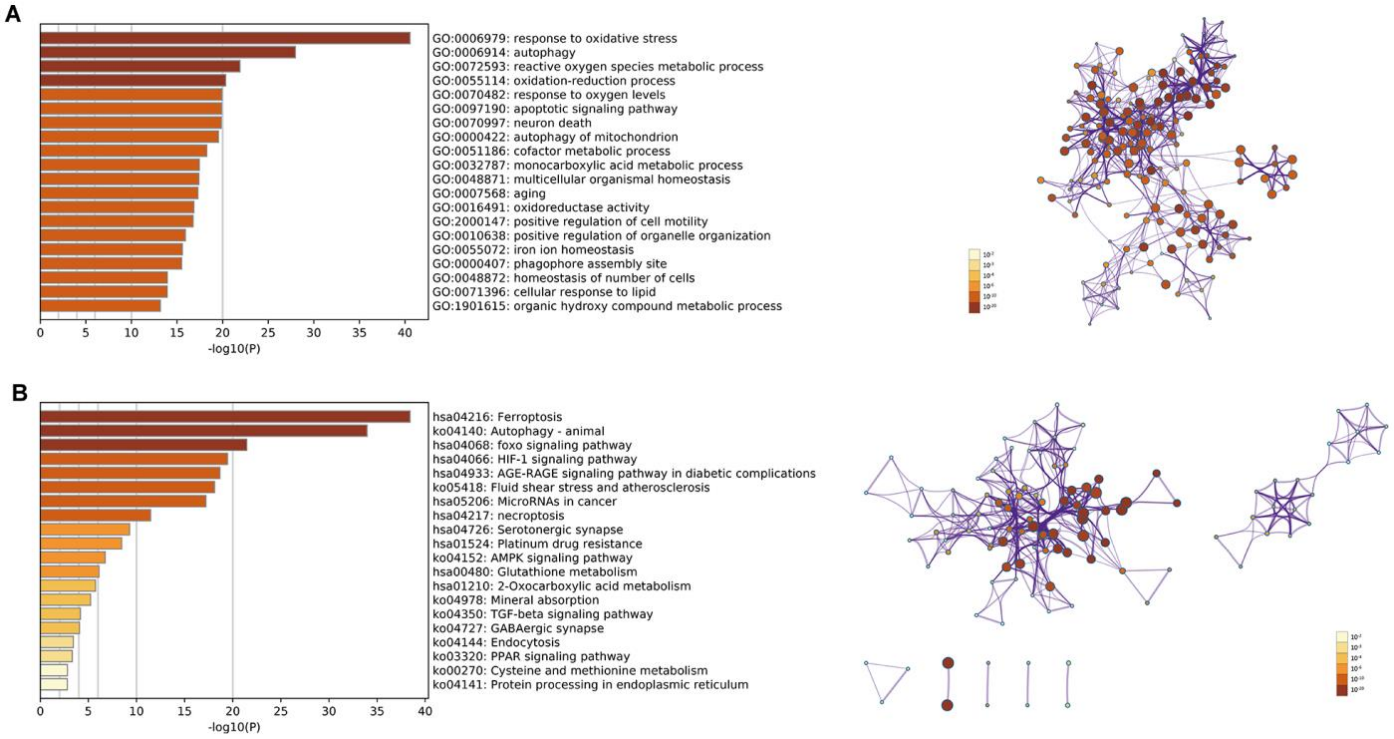
(**A**) Top 20 enriched clusters of DEfrGenes in GO annotation analysis; (**B**) top 20 enriched KEGG pathways of DEfrGenes.

We identified 142 DEfrgenes in COAD tissues (Supplementary Table 8). Based on these genes, we explored the underlying biological functions by GO annotation and KEGG pathway analyses using Metascape. In this study, GO pathway and process enrichment analysis included molecular functions (functional set), biological processes (pathway), and cellular components (structural complex). The top 20 clusters with their representative enriched terms are shown in Figure 6A. Our results showed that biological processes related to oxygen metabolism were significantly associated with differentially expressed ferroptosis-related genes, including GO:0006979 (response to oxidative stress), GO:0072593 (reactive oxygen species metabolic process), GO:0055114 (oxidation-reduction process), GO:0070482 (response to oxygen levels), GO:0016491 (oxidoreductase activity), and GO:1901615 (organic hydroxy compound metabolic process). In addition, autophagy-related biological processes, such as GO:0006914 (autophagy), GO:0000422 (autophagy of mitochondria), GO:0055072 (iron ion homeostasis), and GO:0000407 (phagophore assembly site) were prominently related to differentially expressed ferroptosis-related genes. Moreover, DEfrGenes in COAD also dramatically affected GO:0097190 (apoptotic signaling pathway), GO:0070997 (neuron death), and several metabolism-related biological processes.

The top 20 KEGG pathways (*P*<0.05) for the DEfrGenes were identified (Figure 6B). Classical cancer-related pathways such as “foxo signaling pathway,” “MicroRNAs in cancer,” “necroptosis,” “platinum drug resistance,” “AMPK signaling pathway,” “TGF-beta signaling pathway” and “PPAR signaling pathway” were correlated with the functions of the DEfrGenes in COAD. It is worth noting that “HIF-1 signaling pathway” was the fourth most significantly correlated signaling pathway.

### Correlations between the risk score model and hypoxia-related factors

**Figure 7:**
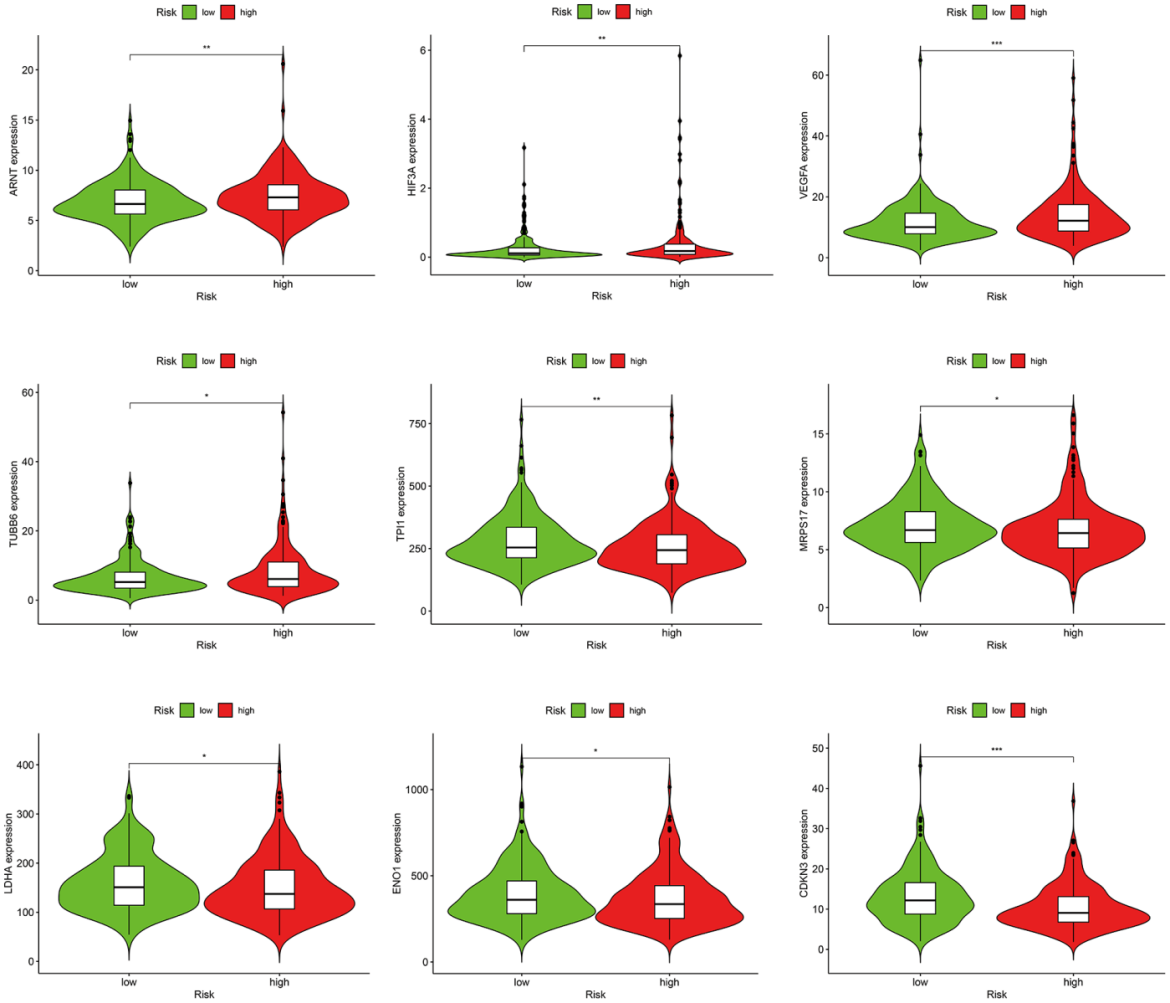
Correlations between the risk score model and hypoxia-related factors.

GO and KEGG analysis showed that frGenes were notably related to biological processes of oxygen metabolism. Therefore, we further investigated the relationship between the risk score model and hypoxia-related factors, including hypoxia-inducible factors, and hypoxia-related genes (Supplementary Table 9). The results revealed that the high-risk group was significantly correlated with high expression of ARNT, HIF3A, VEGFA, TUBB6, and low expression of TPI1, MRPS17, LDHA, ENO1, and CDKN3 (Figure 7).

### Correlations between the risk score model and immune-related factors

**Figure 8:**
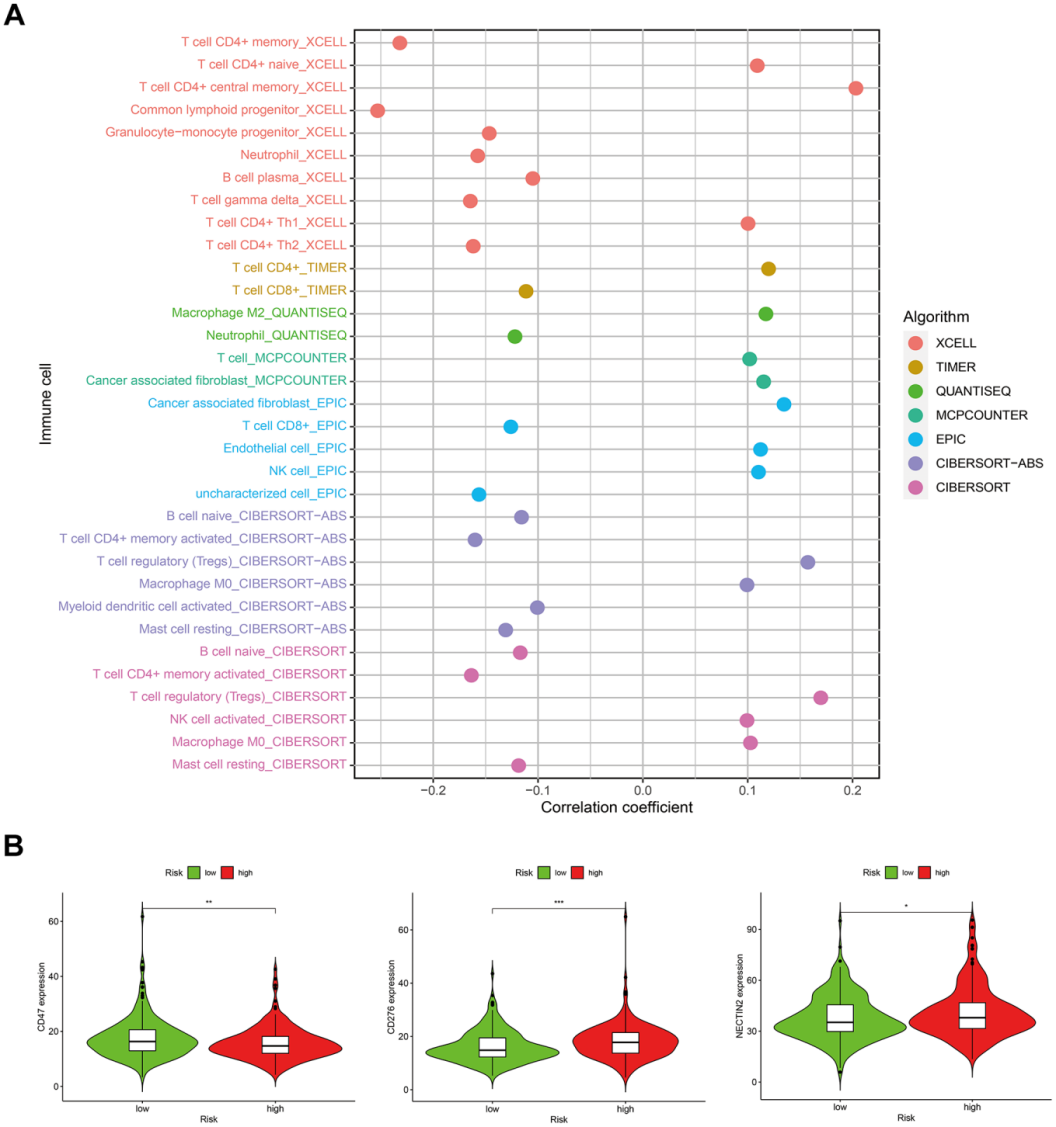
Correlations between the risk score model and (**A**) tumor-infiltrating immune cells; (**B**) immune checkpoint genes.

In consideration of the increasing evidence on the correlation between immunological features and survival in malignant tumors, we discussed the correlations between the risk score model and immune-related factors. The immune-related factors included tumor-infiltrating immune cells (TIICs) and immune checkpoint genes (ICGs). The 16 ICGs were chosen based on a previous study by Danilova [25] with minor modifications by adding ligands (PVR and NECTIN2) and competing receptor (CD226) of TIGIT (Supplementary Table 10) [26, 27]. We discovered that the low-risk group was related to more TIICs such as CD8+ T cells, CD4+ T cells, B cells, and neutrophils, whereas the high-risk group was related to more tumor-infiltrating immune cells such as NK cells, macrophages, and T cell regulatory (Tregs) (Figure 8A). In addition, the high-risk group had significantly downregulated expression of CD47 and markedly upregulated expression of CD276 and NECTIN2 (Figure 8B).

### Correlations between the risk score model and somatic variants

**Figure 9:**
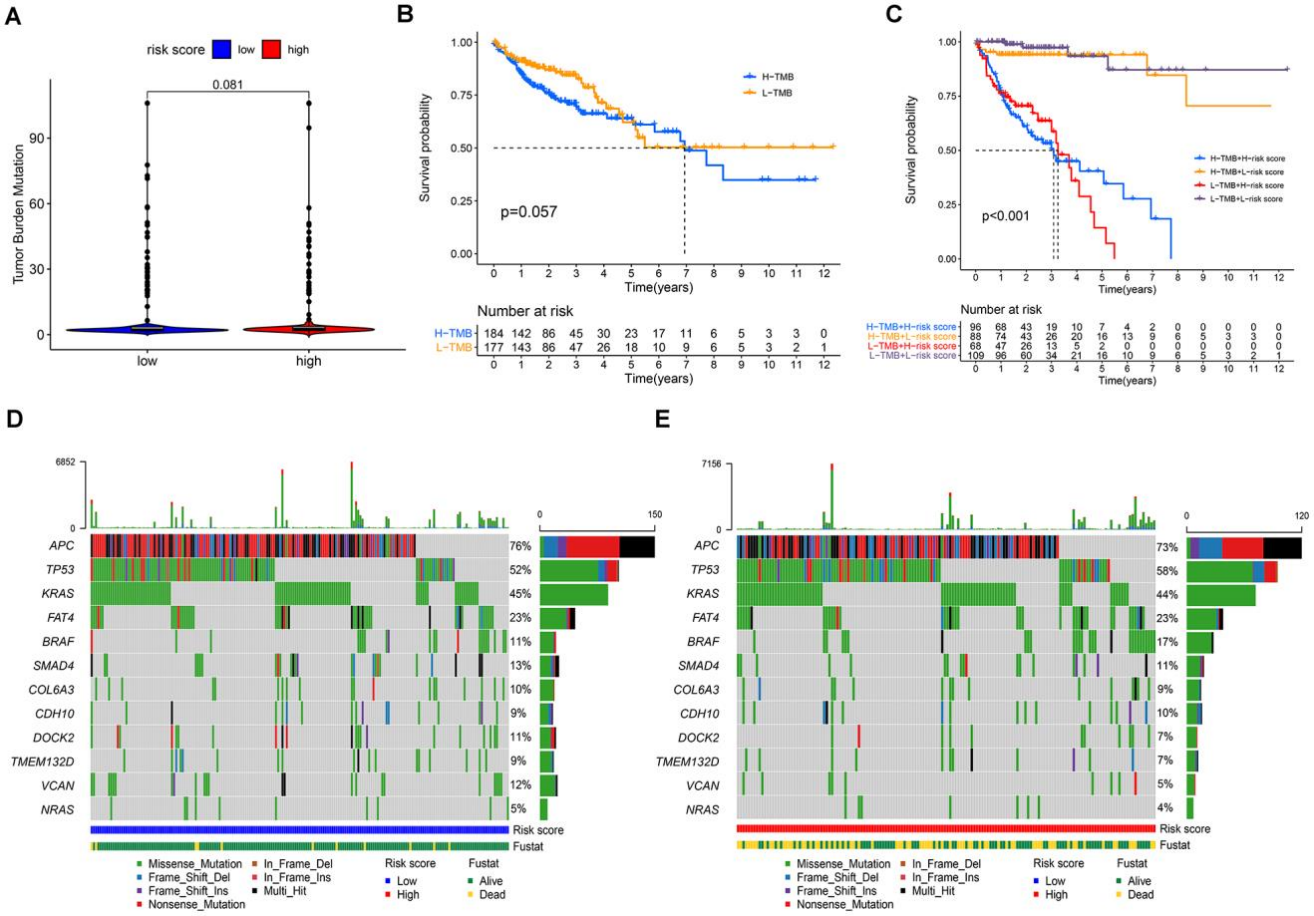
**Correlations between the risk score model and somatic variants.** (**A**) TMB levels between the high- and low-risk groups; (**B**) correlation between TMB and prognosis in patients with COAD; (**C**) prognostic predictive value in different TMB subgroups; (**D**, **E**) the mutation rates of reported prognostic-related genes in low- and high-risk groups.

Studies have shown that cancers harboring more non-synonymous variants, which were defined as high tumor mutation burden (TMB), were associated with favorable survival outcomes with cancer immunotherapy. Given the prognostic value of TMB, we sought to investigate the relationship between the risk score model and TMB level. As shown in Figure 9A, there was no significant difference in TMB levels between the high- and low-risk groups. Furthermore, our study found no significant correlation between TMB and prognosis in patients with COAD (Figure 9B). Next, we evaluated the predictive power of the risk score model in the low and high TMB subgroups. The results revealed that the prognostic model presented consistent predictive value in both the low and high TMB subgroups, indicating that the TMB status did not interfere with the prediction of this model (Figure 9C). Moreover, our study explored the mutation rates of reported prognostic-related genes in low-and high-risk groups. Analysis results demonstrated that APC, SMAD4, DOCK2, TMEM132D, and VCAN genes had more frequent mutations in the low-risk group, whereas TP53 and BRAF mutations were more frequent in the high-risk group (Figure 9D, 9E).

### Gene set enrichment analysis (GSEA)

**Figure 10:**
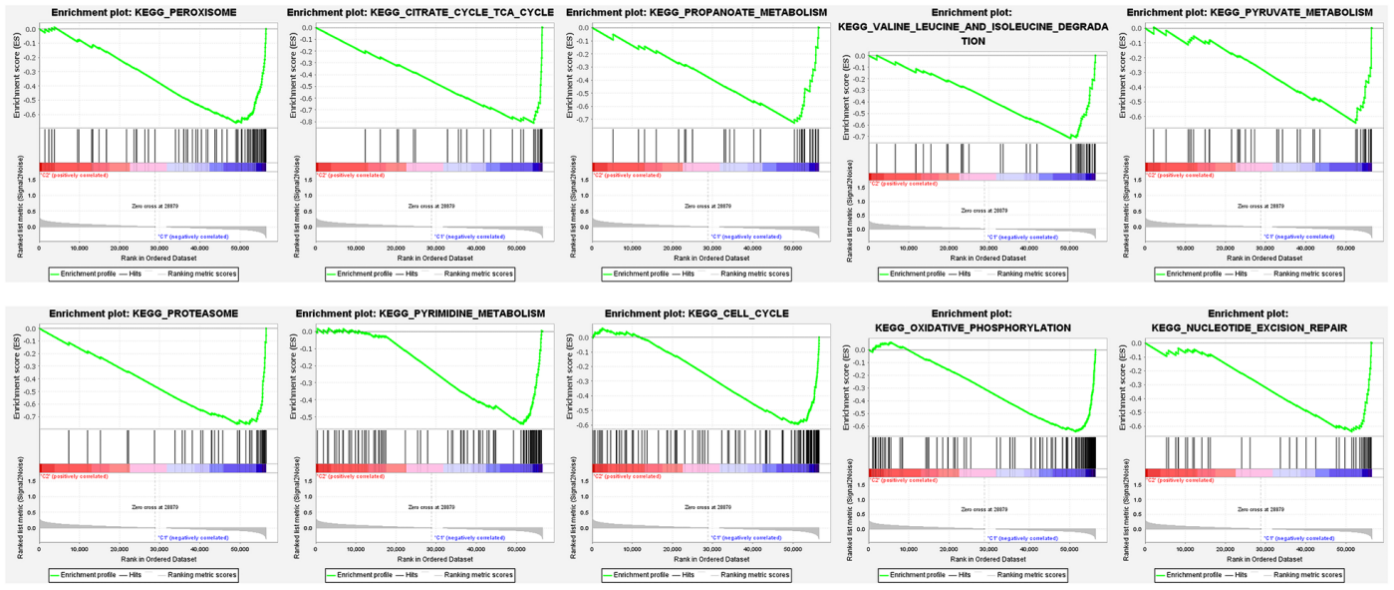
Gene set enrichment analysis of enriched signaling pathways.

The GSEA results indicated that the high-risk score group had markedly negative correlations with 26 enrichment pathways (Supplementary Table 11). As showed in Figure 10, the top 10 KEGG pathways included “peroxisome,” “citrate cycle,” “cell cycle,” “oxidative phosphorylation,” “nucleotide excision repair,” and metabolism-related signaling pathways such as “propanoate metabolism,” “valine leucine and isoleucine degradation,” “pyruvate metabolism,” “pyrimidine metabolism,” and so on.

## DISCUSSION

Ferroptosis is an iron-dependent cell death, which is characterized by an intracellular iron-dependent accumulation of ROS and lipid peroxidation. Shen et al. found resibufogenin inhibited colorectal cancer cell growth and tumorigenesis through triggering ferroptosis, suggesting that suppression of ferroptosis is closely related to the proliferation of colorectal cancer cells [28]. Sui and colleagues demonstrated that RSL3-induced cell ferroptosis was relevant to colorectal cancer progression [29]. They revealed that RSL3 can promote CRC cell ferroptosis by promoting ROS production. Sujeong Park et al. reported that bromelain effectively inhibited cell growth and proliferation by stimulating ferroptosis, especially in Kras mutant colorectal cancer cells [30]. The findings indicate that ferroptosis may be involved in carcinogenesis in mutant colorectal cancer cells, compared to Kras wild-type colorectal cancer cells. These studies showed that ferroptosis is crucial in regulating the growth and proliferation of colon cancer cells, and drug activation of ferroptosis related signaling pathways is a promising strategy for treating colon cancer. Moreover, studies have shown that ferroptosis related gene signatures can be effectively used for prognostic prediction, optimizing survival risk assessment and facilitating personalized management in colon cancer [31–33]. These results indicate that the research of ferroptosis can provide ideas for the clinical diagnosis and treatment of colon cancer. LncRNA prognostic signatures have been reported to have promising predictive value in cancer. In recent years, prognostic prediction models based on specific classes of gene-related lncRNAs have attracted particular attention from researchers. However, most of these prognostic models have limited predictive efficacy. For example, the AUC was 0.782 for a signature that included 25 differentially expressed ferroptosis-related lncRNAs in predicting the prognosis of HNSCC [19]. An autophagy-related lncRNA signature had an AUC value of 0.689, which comprised eight lncRNAs and predicted an unfavorable prognosis in colorectal cancer [22]. A prognostic model including four differentially expressed lncRNAs had an AUC value of 0.706 for evaluating the outcome of patients with colorectal cancer [23]. In addition to unsatisfactory predictive performance, these models also have practical shortcomings. These prognostic models were established based on the specific expression values of the identified lncRNAs. The measured values must be normalized to reduce batch effects between different testing platforms before clinical application. In the present study, we developed a novel ferroptosis-related lncRNA pair prognostic signature for colon adenocarcinoma. The AUCs representing the excellent predictive value for 1-, 3-, and 5-year survival rates were 0.860, 0.885, and 0.934, respectively. Our prognostic signature included 25 frlncRNA pairs and was confirmed as an independent prognostic factor by Cox regression analysis. Notably, it was superior to common clinicopathological characteristics such as age, sex, T status, N status, and M status in predicting the OS for COAD. More importantly, the signature was established by a novel modeling algorithm, pairing, and iteration; therefore, it could be better applied in clinical practice.

To explore the underlying biological functions of our signature, we performed GO annotation and KEGG pathway analyses of the identified DEfrGenes. Both function and pathway enrichment analyses highlighted the hypoxia and oxygen metabolism processes, such as “HIF-1 signaling pathway,” “response to oxidative stress,” “reactive oxygen species metabolic process,” “oxidation-reduction process,” “response to oxygen levels,” “oxidoreductase activity” and “organic hydroxy compound metabolic process.” Studies have found that hypoxia is a poor prognostic factor that regulates the tumor microenvironment. Hypoxia activates a series of signaling pathways and a large panel of genes involved in apoptosis, autophagy, DNA damage, mitochondrial activity, p53, and drug efflux, which affects the survival of cancer cells and confers resistance to conventional anticancer treatments [34, 35]. Preclinical studies have shown that inhibition of HIF-1 signaling activity can significantly reduce cancer growth, and research is currently underway to identify HIF-1 inhibitors and validate their efficacy in antitumor therapy [36]. Several hypoxia-related signatures have been constructed for the clinical prediction of diagnosis, prognosis, and recurrence in hepatocellular carcinoma. These hypoxia-related signatures were revealed to be closely associated with the immune microenvironment, contributing to better clinical management in patients with hepatocellular carcinoma [37–39].

For the hypoxia and oxygen metabolism processes, as indicated by both the GO and KEGG analyses, we further explored the relationship between the risk score model and hypoxia-inducible factors and hypoxia-related genes. The results revealed that the high-risk group exhibited a significant correlation with high expression of ARNT, HIF3A, VEGFA, TUBB6 and low expression of TPI1, MRPS17, LDHA, ENO1, and CDKN3. Studies have demonstrated that high levels of HIF-1/2α proteins are associated with increased radiotherapy and chemotherapy resistance, resulting in cancer progression [40, 41]. In addition to inducing hypoxia, the HIF signaling pathway also participates in various bioregulatory processes, including ROS, cytokines, growth factors, oncogene dysfunction, and tumor suppressors, providing insights into the targeting of the HIF signaling pathway as a potential therapeutic approach in cancer management [42]. Studies have reported that hypoxia can promote disease development and metastasis by activating the HIF1α/VEGFA pathway in breast and colorectal cancer [43, 44]. Liang and colleagues found that hypoxia activated the HIF1α/VEGFA axis in breast cancer angiogenesis by inducing miR-153 expression and decreased expression of HIF1α and VEGFA, resulting in suppression of tumor angiogenesis. Chen et al. demonstrated that hypoxia could induce angiogenesis in colorectal cancer by activating the HIF-1α/VEGF-A pathway. LDHA and ENO1 are crucial glycolytic enzymes, which have been revealed as hypoxia-related factors in previous studies. Wei et al. found that inhibition of LDHA-mediated aerobic glycolysis could markedly suppress the growth of bladder cancer cells [45]. Cui et al. reported that HIF1/2α could activate LDHA expression, and high expression of LDHA promotes the growth and migration of pancreatic cancer cells [46]. Nevertheless, Wang et al. reported that high LDHA expression was associated with better PFS (18.3 vs. 10.1 months, *P*=0.005) and overall response rate (72.2% vs. 15.4%, *P*=0.006) of metastatic colorectal cancer patients receiving first-line chemotherapy [47]. Wang et al. discovered the role of ENO1 in hypoxia-induced gemcitabine chemoresistance, decreased expression of which restored sensitivity to chemotherapy by modulating redox homeostasis in pancreatic cancer cells [48]. However, our results showed lower expression of LDHA and ENO1 in the high-risk score group, which suggests some unknown underlying signal regulation mechanisms, which are worth further exploration.

The immune microenvironment plays an important role in tumorigenesis. Infiltrating immune cells may act as tumor-antagonizing or tumor-promoting factors [49, 50]. Cancer cells eventually gain the ability to inhibit the tumor-antagonizing functions of immune cells and escape immunological surveillance, resulting in cancer development [51]. In recent years, immunotherapy targeting immune microenvironment regulation and immune checkpoint modulation has shown promising efficacy in cancer treatment [52, 53]. In addition, iron and immunity are closely linked [54]. Many of the genes and proteins involved in iron homoeostasis play a vital role in controlling iron fluxes. Cells of the innate immune system, monocytes, macrophages, microglia and lymphocytes, are able to combat bacterial insults by carefully controlling their iron fluxes, which are mediated by hepcidin and ferroportin. The cell involved in iron overload with the greatest effect on immunity is the macrophage. Intriguing evidence indicated that parenchymal iron overload is linked to genes classically associated with the immune system [55]. In the present study, we investigated the correlation between the risk score model and immune-related factors. The results showed that the low-risk group was related to more tumor-infiltrating immune cells such as CD8+ T cells, CD4+ T cells, B cells, and neutrophils, whereas the high-risk group was related to more tumor-infiltrating immune cells such as NK cells, macrophages, and Tregs. Moreover, our analyses showed that the high-risk group had significantly decreased expression of CD47 and markedly upregulated expression of CD276 and NECTIN2. Studies have shown that CD47 plays a key role in immune regulation and tumor development. Overexpression of CD47 could protect tumor cells from phagocytosis and is a promising therapeutic target in cancer therapy [56, 57]. CD276, also known as B7-H3, is an important immune checkpoint member of the B7 and CD28 families. CD276 was found to be overexpressed in various tumor cells, which acts as an inhibitor of T cell function and indicates poor prognosis in cancer patients [58, 59]. Studies have shown that NECTIN2 is an adhesion molecule that participates in tumor growth, metastasis, and tumor angiogenesis [60]. There were significant differences in tumor-infiltrating immune cells and ICGs between the high-risk and low-risk groups in our prognostic signature, which may serve as potential molecular markers for predicting the efficacy of immunotherapy.

TMB is not only a prognostic predictor, but also a predictor of the efficacy of immunotherapy. TMB has been demonstrated to be a useful biomarker for predicting the efficacy of immune checkpoint inhibitors in some cancer types [61]. Studies have shown a positive association between TMB and the response to immunotherapy in melanoma and NSCLC [62, 63]. Cao et al. found that patients with high TMB may benefit more from immunotherapy and experience better survival time in various cancer types [64]. However, the predictive role of TMB in the prognosis and efficacy of immunotherapy in colon cancer remains controversial. Lee et al. revealed that high TMB indicated better outcomes for patients treated with curative surgery followed by adjuvant fluoropyrimidine and oxaliplatin chemotherapy [65]. In contrast, Pai et al. found that the low TMB group had improved progression-free survival time (9.9 months) compared to the intermediate/high TMB group (5.8 months) in metastatic colorectal cancer patients treated with first-line chemotherapy [66]. Zhou et al. constructed a nomogram model for prognosis prediction in CC patients, which combined TMB profiles, immunocyte infiltration status, and clinicopathological data. The results showed that patients with low TMB had better outcomes [67]. Indeed, the prognostic value of TMB and its predictive effect on immunotherapy are controversial. Recently, a study found that TMB could only predict response to immunotherapy in cancer types where CD8 T cell levels positively correlated with neoantigen load, such as melanoma, lung, and bladder cancers [68]. The present study showed that TMB was not a significant prognostic factor for CC. There was no significant difference in TMB levels between the high- and low-risk groups. Furthermore, our study suggests that the prognostic role of the signature is independent of TMB levels. We further discovered that the high-risk group had more frequent mutations in TP53 and BRAF, as well as less frequent mutations in APC, SMAD4, DOCK2, TMEM132D, and VCAN genes. Studies have shown that TP53 mutations may accelerate the progression of CRC by activating oncogenic and inflammatory pathways [69]. BRAF mutation was an independent factor of recurrence in microsatellite-stabilized colon cancer, and the outcome of CC patients with BRAF gene mutations was significantly poor [70]. Studies have reported that APC mutation is the most common mutation, accounting for 60% of gene mutations in CC [71]. The current results confirm the role of these genes in predicting prognosis in patients with colon cancer, which is consistent with a previous study [72].

The present study establishes a novel ferroptosis-related lncRNA pair prognostic model that may provide better management in patients with COAD. However, some limitations still need to be considered. First, we only draw conclusions from bioinformatics analysis, and further experimental verification is required. Second, due to the small sample size, there are some statistical defects, for example, the hazard ratio of this the risk score model has a big error range in univariate and multivariate analysis.

## CONCLUSIONS

The novel risk score model constructed by pairing DEfrlncRNAs showed promising clinical prediction value in COAD, which is worthy of further investigation.

## MATERIALS AND METHODS

### Data collection

High-throughput sequencing (HTSeq) transcriptome profiling harmonized to fragments per kilobase million (FPKM) and simple nucleotide variation data of COAD as well as clinical characteristics of patients were obtained from The Cancer Genome Atlas (TCGA) database (https://portal.gdc.cancer.gov). Cases with survival data were randomly divided into the training cohort and validation cohort in 2:1 ratio. The GTF file of long non-coding RNA gene annotation (version GRCh38.p13) was downloaded from the GENCODE human website (https://www.gencodegenes.org/human/). The ferroptosis-related genes involved in the current study were obtained from the website of FerrDb, a public database of ferroptosis regulators and markers, and ferroptosis-disease associations (http://www.zhounan.org/ferrdb/) [73].

### Identification of frlncRNAs and pairing DEfrlncRNAs

Identification of the frlncRNAs was performed using Pearson correlation to assess the relationship between the ferroptosis-related genes and long non-coding RNAs. The absolute value of correlation coefficients >0.4 and *P*<0.001 were considered statistically significant. The significant thresholds of DEfrlncRNAs were set as |log2FC| >1.5 and false discovery rate (FDR) <0.001. Subsequently, we established frlncRNA pairs based on these DEfrlncRNAs, as previously described [24]. All the DEfrlncRNAs were cyclically paired and assigned a value according to pairwise comparison in accordance with the following rules: assume that lncRNA A and lncRNA B were paired together as lncRNA pair C, which was assigned a value of 1 if the expression level of lncRNA A was higher than lncRNA B; otherwise, it was assigned a value of 0. If a lncRNA pair had a 0 or 1 ratio of less than 20% or greater than 80% in all samples, it was filtered. Cox regression analyses were performed to evaluate the prognostic value of frlncRNA pairs (*P*<0.01).

### Construction and validation of a frlncRNA pair prognostic signature

Prognostically associated frlncRNA pairs were used to establish the LASSO regression model in the training cohort. Subsequently, a risk score model of these frlncRNA pairs was constructed and the risk score of each patient was calculated according to the following formula: Risk score = coefficient lncRNA pair^1^ × expression lncRNA pair^1^ + coefficient lncRNA pair^2^ × expression lncRNA pair^2^ + coefficient lncRNA pair^3^ × expression lncRNA pair^3^ +……+ coefficient lncRNA pair^n^ × expression lncRNA pair^n^. The 1-year ROC curve for predicting the OS was constructed according to the risk score model. Using the median value of risk scores, patients in training- and validation cohort were divided into high- and low-risk groups. Survival curves were conducted using the Kaplan-Meier method. The validation cohort was applied for cross test to assess the stability of this model. In order to obtain a more accurate model with a larger sample size, we further constructed the final model based on the data of entire cohort. The 1-, 3-, and 5-year ROC curves were conducted. The maximum inflection point of the 5-year ROC curve was considered as the optimal cut-off point for the classification of different risk groups. A risk assessment model for prognosis prediction was generated to show the risk scores and survival outcomes of each patient. Moreover, univariate and multivariate regression analyses were used to evaluate whether the model was an independent prognostic factor for OS in COAD patients.

### Clinical correlation analysis of the risk score model and development of a nomogram

The correlations between the risk score model and traditional clinicopathological features were assessed using the chi-squared test and presented as a heatmap. The risk score differences among different groups of these clinicopathological features were calculated using the Wilcoxon signed-rank test and visualized using box diagrams. The p value was labeled as follows: *P*<0.001 = ***, *P*<0.01 = ** *, and *P*<0.05 = *. The multivariate logistic model, including the risk score model and clinicopathological characteristics, was used to develop a nomogram to predict the survival probability of COAD patients. The 1-, 3-, and 5-year OS rates were used as the endpoints in the nomogram.

### Correlations of the risk score model with hypoxia-related and immune-related factors

DEfrGenes in COAD tissues were identified using the R package limma. Gene Ontology (GO) and Kyoto Encyclopedia of Genes and Genomes (KEGG) analyses of DEfrGenes were performed using Metascape (http://metascape.org) with thresholds of min overlap 3, *P*<0.05, and min enrichment 3 [74]. Hypoxia-inducible factors included HIF1A, ARNT, EPAS1, ARNT2, HIF3A, and ARNTL. Hypoxia-related genes were retrieved from previous reports [75, 76]. Differential gene expression between the two risk score groups was conducted using the Wilcoxon signed-rank test. We employed several currently acknowledged algorithms to evaluate the correlations between the risk score and TIICs, including XCELL, TIMER, QUANTISEQ, MCPCOUNTER, EPIC, CIBERSORT−ABS, and CIBERSORT. Associations between the risk score groups and the expression of ICGs were assessed, and the results were presented as violin plots. The statistical significance was set at *P*<0.05.

### Correlations between the risk score model and somatic variants

To evaluate the TMB, we counted the total number of non-synonymous mutations in COAD. TMB was defined as the number of somatic, coding, indel mutations, and base substitution per megabase (Mb) of the genome examined. To calculate the TMB score of each sample, the total number of mutations counted was divided by the exome size (38 Mb was used as the estimate of the exome size) [77]. The somatic variant status of reported prognostic-related genes in COAD was assessed in low- and high-risk groups. The included genes were APC, TP53, KRAS, NRAS, BRAF, FAT4, SMAD4, COL6A3, CDH10, DOCK2, TMEM132D, and VCANT, which were retrieved from a previous study [72].

### Gene set enrichment analysis

Gene set enrichment analysis (high-risk score group vs. low-risk score group) (version 4.1.0; http://software.broadinstitute.org/gsea/index.jsp) was performed to investigate the potential molecular mechanisms by which the risk score model might act on tumor progression in COAD, as previously described [78, 79]. FDR <0.05 was used to identify significantly enriched pathways.

### Statistical analysis

Perl software (version 5.32) was used to extract and structure the HTSeq FPKM and simple nucleotide variation data. The differentially expressed lncRNAs were identified using the Benjamini-Hochberg method based on the log fold change and FDR. Survival analyses of COAD patients based on the risk score model were assessed using the Kaplan-Meier method. Multivariate analysis was performed using the Cox regression model. R software version 4.0.3, with Bioconductor packages, was used to conduct the analyses.

### Data statement

The data used for bioinformatics analyses in this study are freely available on the National Cancer Institute Genomic Data Commons (GDC) Data Portal (https://portal.gdc.cancer.gov/). The interpretation and reporting of these data are the sole responsibility of the authors.

## Supplementary Material

Supplementary Figures

Supplementary Table 1

Supplementary Table 2

Supplementary Table 3

Supplementary Table 4

Supplementary Table 5

Supplementary Table 6

Supplementary Table 7

Supplementary Table 8

Supplementary Tables 9-11
