# Supplementary material for: Establishment of a novel ferroptosis-related lncRNA pair prognostic model in colon adenocarcinoma
Source: Aging (Albany NY). 2021 Oct 5;13(19):23072–95. doi: 10.18632/aging.203599 (PMC8544324; doi:10.18632/aging.203599)
Supplement: Supplementary Figures [file aging-13-203599-s001.pdf]

SUPPLEMENTARY FIGURES

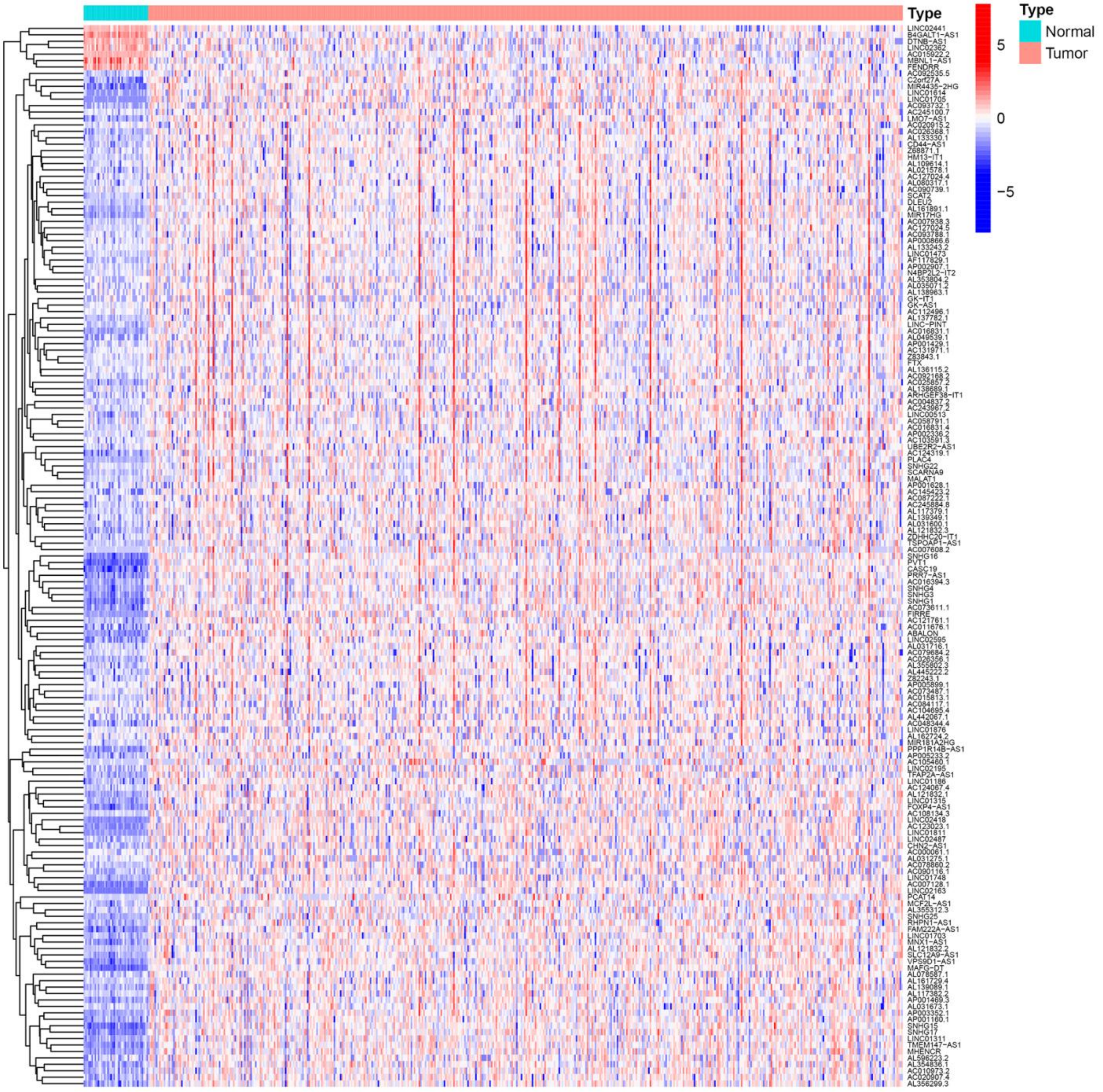

Supplementary Figure 1. Differentially expressed frlncRNAs in COAD visualized by a heatmap.

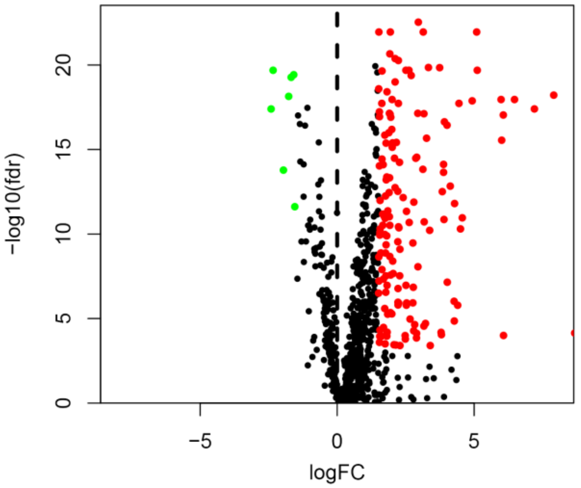

Supplementary Figure 2. Differentially expressed frlncRNAs in COAD represented by a volcano plot.

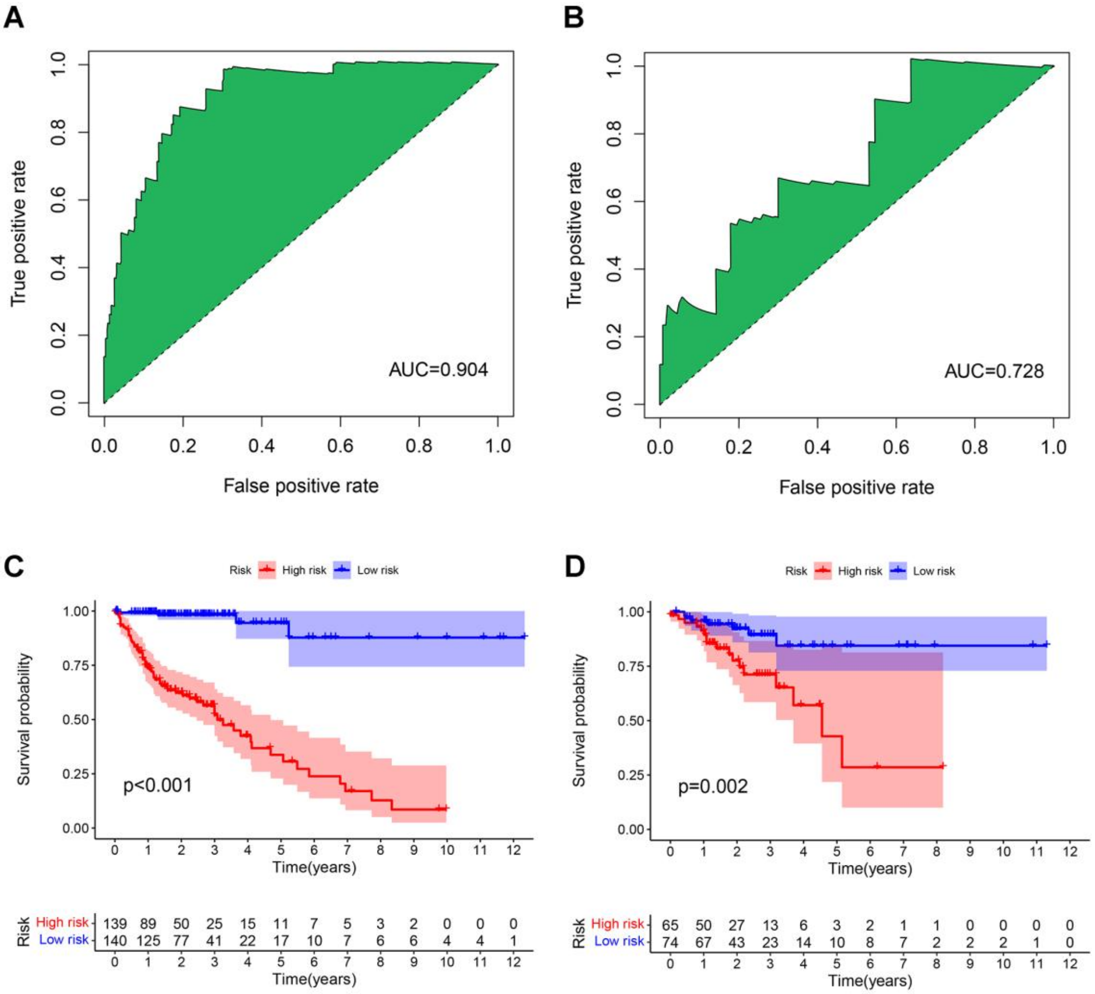

Supplementary Figure 3. The ROC curves for predicting the 1-year OS in (**A**) training cohort and (**B**) validation cohort; survival curves of high-risk and low-risk group patients in (**C**) training cohort and (**D**) validation cohort.
