# Supplementary material for: Establishment of a novel ferroptosis-related lncRNA pair prognostic model in colon adenocarcinoma
Source: Aging (Albany NY). 2021 Oct 5;13(19):23072–95. doi: 10.18632/aging.203599 (PMC8544324; doi:10.18632/aging.203599)
Supplement: Supplementary Table 6 [file aging-13-203599-s007.pdf]

Supplementary Table 6. The list of lncRNA pairs and corresponding calculation coefficients in training cohort.

Gene	Coef
AP000866.6|GK-AS1	0.050517142
LINC01614|AC145423.2	0.325460089
LINC01876|AC123023.1	0.021230585
LINC01876|AC000061.1	0.017454934
LINC01703|FENDRR	0.351506677
B4GALT1-AS1|AL109614.1	-0.470853267
LINC02487|AC010973.2	-0.44273071
ARHGEF38-IT1|AL136115.2	-0.090659931
LINC02195|AC048344.4	-0.168551255
AC020907.4|AC010973.2	-0.128374285
MHENCR|AC025857.2	0.14001933
AL031716.1|AL117379.1	-0.026252276
AL031716.1|AC245884.8	-0.048075783
MIR17HG|AL161729.4	-0.201226884
AC127024.4|AL355802.3	0.247392389
AC000061.1|AC007938.3	-0.076967626
AC000061.1|AL445222.2	-0.166595929
LINC01748|LINC00513	0.239026862
AP005233.2|SLC12A9-AS1	0.013512242
AC010973.2|AP002336.2	0.000819065
AC010973.2|LINC01311	0.009915108
AC010973.2|SCARNA9	0.067640044
AC010973.2|AC026356.1	0.024730177
AC010973.2|AL031673.1	0.091362139
TSPOAP1-AS1|AC245100.7	-0.025201899
AC007128.1|ABALON	0.141543495
AL021578.1|AL133243.2	0.307784852
AC092535.5|FENDRR	0.072656736
AP002336.2|AC093732.1	-0.034956361
MIR181A2HG|AC103591.3	0.06281789
AC011676.1|AC092168.2	0.335286777
AC121761.1|AL109614.1	-0.05947878
AC121761.1|CD44-AS1	-0.070098552
AP005899.1|GK-AS1	0.194484479
LINC-PINT|LINC00513	0.376302947
AF117829.1|SNHG22	-0.451862499
AC245100.7|LINC01811	0.228863795
SCARNA9|AC104695.4	-0.074535782
HM13-IT1|MAFG-DT	0.096156896
AL137782.1|AC245884.8	-0.184358171
AL137782.1|AP001469.3	-0.46691788
AL161729.4|AL031673.1	0.03850454
GK-AS1|AC084117.1	-0.12372433
ABALON|CD44-AS1	-0.066789815
