# Supplementary material for: Establishment of a novel ferroptosis-related lncRNA pair prognostic model in colon adenocarcinoma
Source: Aging (Albany NY). 2021 Oct 5;13(19):23072–95. doi: 10.18632/aging.203599 (PMC8544324; doi:10.18632/aging.203599)
Supplement: Supplementary Tables 9-11 [file aging-13-203599-s010.pdf]

Supplementary Table 9. The list of hypoxia-related genes.

Gene
VEGFA
SLC2A1
PGAM1
ENO1
LDHA
TPI1
P4HA1
MRPS17
CDKN3
ADM
NDRG1
TUBB6
ALDOA
MIF
ACOT7

Supplementary Table 10. The list of immune checkpoint genes.

Gene
CD274
CTLA4
IDO1
LAG3
CD276
VTCN1
CD70
HAVCR2
CD40
CD47
TNFRSF18
TNFSF14
TIGIT
PVR
NECTIN2
CD226

Supplementary Table 11. Cancer-related Kyoto Encyclopedia of Genes and Genomes (KEGG) pathways associated with risk group based on a gene set enrichment analysis.

	Enrichment in phenotype: C1<br>GS	ES	NES	NOM p-val	FDR q-val	FWER p-val
1	KEGG_PEROXISOME	-0.66	-2.24	0	0.001	0.001
2	KEGG_CITRATE_CYCLE_TCA_CYCLE	-0.81	-2.23	0	0	0.001
3	KEGG_PROPANOATE_METABOLISM	-0.73	-2.15	0	0.003	0.009
4	KEGG_HUNTINGTONS_DISEASE	-0.58	-2.14	0	0.002	0.01
5	KEGG_ALZHEIMERS_DISEASE	-0.57	-2.12	0	0.003	0.013
6	KEGG_VALINE_LEUCINE_AND_ISOLEUCINE_DEGRADATION	-0.72	-2.05	0.002	0.007	0.028
7	KEGG_PYRUVATE_METABOLISM	-0.64	-2.04	0	0.007	0.031
8	KEGG_PROTEASOME	-0.76	-2.04	0	0.007	0.035
9	KEGG_PARKINSONS_DISEASE	-0.63	-1.98	0.008	0.012	0.061
10	KEGG_PYRIMIDINE_METABOLISM	-0.54	-1.97	0.008	0.014	0.073
11	KEGG_CELL_CYCLE	-0.59	-1.95	0.012	0.014	0.079
12	KEGG_OXIDATIVE_PHOSPHORYLATION	-0.64	-1.94	0.01	0.013	0.083
13	KEGG_OOCYTE_MEIOSIS	-0.52	-1.94	0.002	0.013	0.088
14	KEGG_NUCLEOTIDE_EXCISION_REPAIR	-0.64	-1.91	0.01	0.016	0.115
15	KEGG_BUTANOATE_METABOLISM	-0.61	-1.85	0.008	0.026	0.174
16	KEGG_FATTY_ACID_METABOLISM	-0.59	-1.84	0.01	0.031	0.211
17	KEGG_CYSTEINE_AND_METHIONINE_METABOLISM	-0.54	-1.84	0.004	0.029	0.214
18	KEGG_UBIQUITIN_MEDIATED_PROTEOLYSIS	-0.5	-1.83	0.013	0.028	0.217
19	KEGG_PROTEIN_EXPORT	-0.72	-1.82	0.014	0.029	0.227
20	KEGG_AMINOACYL_TRNA_BIOSYNTHESIS	-0.65	-1.82	0.021	0.029	0.239
21	KEGG_PURINE_METABOLISM	-0.43	-1.8	0.008	0.032	0.262
22	KEGG_GLUTATHIONE_METABOLISM	-0.52	-1.8	0.002	0.031	0.266
23	KEGG_BIOSYNTHESIS_OF_UNSATURATED_FATTY_ACIDS	-0.62	-1.78	0.01	0.034	0.3
24	KEGG_DNA_REPLICATION	-0.72	-1.77	0.02	0.037	0.323
25	KEGG_BASAL_TRANSCRIPTION_FACTORS	-0.59	-1.77	0.023	0.036	0.325
26	KEGG_MISMATCH_REPAIR	-0.69	-1.73	0.018	0.049	0.401
